# A Prognostic Model Generated from an Apparent Diffusion Coefficient Ratio Reliably Predicts the Outcomes of Oral Tongue Squamous Cell Carcinoma

**DOI:** 10.3390/curroncol29120708

**Published:** 2022-11-22

**Authors:** Lingling Cai, Xiaoguang Li, Lizhong Wu, Bocheng Wang, Mingjue Si, Xiaofeng Tao

**Affiliations:** 1 Department of Radiology, Shanghai Ninth People’s Hospital, Shanghai Jiao Tong University School of Medicine, Shanghai 201999, China; 2 Department of Oral Maxillofacial-Head and Neck Oncology, Shanghai Ninth People’s Hospital, College of Stomatology, Shanghai Jiao Tong University School of Medicine, National Clinical Research Center for Oral Disease, Shanghai 201999, China; 3 Department of Otolaryngology-Head and Neck Surgery, Shanghai Ninth People’s Hospital, Shanghai Jiao Tong University School of Medicine, Shanghai 200011, China

**Keywords:** tongue cancer, prognostic model, apparent diffusion coefficient, recurrence, disease-free survival

## Abstract

This study aimed to develop an apparent diffusion coefficient (ADC) ratio-based prognostic model to predict the recurrence and disease-free survival (DFS) of oral tongue squamous cell carcinoma (OTSCC). A total of 188 patients with cT1-2 oral tongue squamous cell carcinoma were enrolled retrospectively. Clinical and laboratory data were extracted from medical records. The ADC values were measured at the regions of interest of the tumor and non-tumor tissues of the MRI images, and the ADC ratio was used for comparison between the patient with recurrence (*n* = 83 case, 44%) and patients without recurrence (*n* = 105 cases, 56%). Cox proportional hazards models were generated to analyze the risk factors of cancer recurrence. A nomogram was developed based on significant risk factors to predict 1-, 5- and 10-year DFS. The receiver operator characteristic (ROC) curves of predictors in the multivariable Cox proportional hazards prognostic model were generated to predict the recurrence and DFS. The integrated areas under the ROC curve were calculated to evaluate discrimination of the models. The ADC ratio, tumor thickness and lymph node ratio were reliable predictors in the final prognostic model. The final model had a 71.1% sensitivity and an 81.0% specificity. ADC ratio was the strongest predictor of cancer recurrence in prognostic performance. Discrimination and calibration statistics were satisfactory with C-index above 0.7 for both model development and internal validation. The calibration curve showed that the 5- and 10-year DFS predicted by the nomogram agreed with actual observations.

## 1. Introduction

Oral tongue squamous cell carcinoma (OTSCC) is one of the most common oral cavity cancers with an estimated 8000 annual cases in China [1]. The primary treatment strategy for OTSCC is surgical excision. Multimodality treatments including neck dissection and adjuvant radio- or chemo-therapy are also required for OTSCC with aggressive behavior [2]. Despite recent advances in the diagnosis and the treatments, the prognosis of the OTSCC is poorer than other oral cavity subsite cancers because the cancer cells are more aggressive, easily metastasize and frequently recur [3]. Therefore, precise assessment of the OTSCC clinical behavior and prediction of recurrence prior to the surgery is critical for the development of a personalized therapeutic strategy to improve patient outcomes. While genomic biomarkers identified by sequencing a patient’s entire genome DNA are widely used tools that oncologists use to predict the patient’s outcomes and plan their post-operational treatments, the strategy is expensive and requires a physical sample of the cancer tissue. The recent development of the computational prognostic model using radiological image features and tumor characteristics extracted from medical records of patients with cancer provides a cost-effective, non-invasive approach to predict the risk of recurrence and make a posttreatment monitoring plan [4,5].

Magnetic resonance imaging (MRI) has been routinely used to evaluate clinical behavior of tongue carcinoma [6]. It has another distinctive function of diffusion-weighted imaging (DWI) that can measure the random Brownian motion of water molecules within a voxel of tissue and as such can evaluate the pathologic changes. Highly cellular tissues or swollen tissues exhibit lower diffusion coefficients, which can be quantified by the apparent diffusion coefficient (ADC) [7]. The ADC value is particularly useful in tumor characterization. Tumors with high stromal content and micronecrosis, two biological factors known to be associated with poor prognosis, display abnormal restricted diffusion, resulting in an increased ADC value [8]. Recent studies demonstrated that head and neck squamous cell carcinomas with increased ADC value in MRI responded poorly to radiotherapy and the overall outcome was also poor [9,10,11,12,13]. However, whether ADC values extracted from MRI images can be used to build a prognostic model to predict the OTSCC recurrence and DFS is unknown.

It is known that the demographic characteristics of patients as well as the technical issues of MRI imaging affect the performance of ADC value for assessment of aggressive cancers [14]. To improve performance and overcome the variability, Woo and Siegel used the ADC ratio and showed a relatively better performance than absolute ADC values in patients with prostate cancer [15,16]. These studies suggested that the ADC ratio values extracted from the MRI images would be a better predictor and could be integrated into a multivariable prognostic model for better performance of OTSCC outcome prediction. In this study, we performed a retrospective study to develop an ADC ratio-based prognostic model to predict the recurrence and DFS of OTSCC.

## 2. Materials and Methods

### 2.1. Study Population and Design

A retrospective cohort study was designed to develop the prediction model. The study was approved by the Ethics Review Board of our hospital (Shanghai, China) (Approval No:SH9H-2021-T486-1), and the written informed consent was waived due to the retrospective nature of the study. This study was performed according to the guidelines of the Transparent Reporting of a Multivariable Prediction Model for Individual Prognosis or Diagnosis (TRIPOD).

### 2.2. Inclusion and Exclusion Criteria of Patients

**Figure 1:**
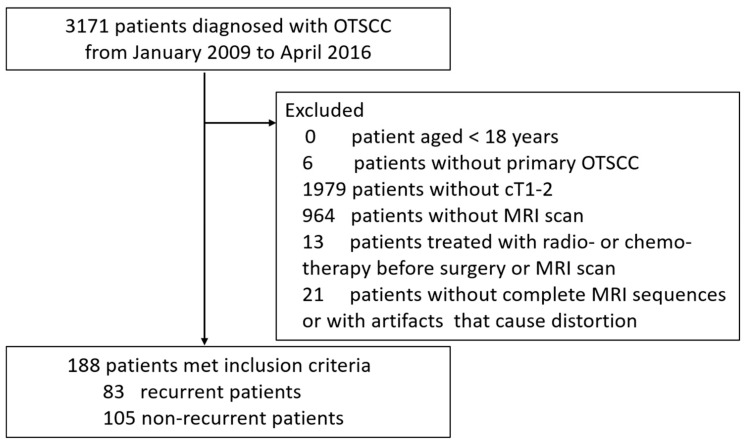
Patient exclusion flowchart.

A total of 188 patients with OTSCC who received surgery at our hospital from January 2009 to April 2016 and met all of the following criteria were enrolled to this study: (1) age ≥ 18 years old, staged as cT1-2 by histopathologists (e.g., the tumor is 2 cm or smaller as cT1, the tumor is larger than 2 cm but not larger than 4 cm as cT2 according to the 8th Edition TNM Classification for Head and Neck Cancer); (2) patients who did not receive any treatments or biopsy procedures before this surgery; (3) patients who completed contrast-enhanced MRI scan within 2 weeks prior to surgery (the index date was defined as the date of the surgery); (4) those who received tumor resection and elective neck dissection during the same period; (5) patients with complete medical records, including the demographics, tumor characteristics, surgical outcomes, and postoperative adjuvant treatments. Patients with postoperative radiotherapy, chemotherapy or concurrent chemo/radiotherapy could be included in the study if they were pathologically diagnosed with lymph nodes or distant metastasis. All patients who received radiotherapy or chemotherapy prior to surgery and their MRI scan were excluded. All patients with incomplete MRI sequences or artifacts that could cause distortion of the tumor were also excluded. As shown in Figure 1, a total of 2983 OTSCC patients were excluded from the study.

### 2.3. MR Imaging Verification and Interpretation

MRI examinations were performed with a 1.5-T scanner (Signa Excite; GE Medical Systems, Milwaukee, WI, USA). The conventional MRI sequences included axial T1 weighted imaging (T1WI) [repetition time (TR)/echo time (TE), 400–600/10 ms], axial and coronal T2 weighted imaging (T2WI) (TR/TE, 3200/100 ms), field of view (FOV) = 240 × 240 mm, matrix = 256 × 192, and thickness/gap, 5/1 mm. DW-MR imaging examination was carried out by using echoplanar single-shot spin-echo sequence (TR/TE = 2775/70 ms; matrix, 128 × 128 mm; FOV, 240 × 240 mm; thickness/gap, 5.0/0.5 mm), with b values of 0, 800s/mm^2^. The ADC maps were automatically generated. Dynamic contrast enhanced MR imaging was obtained after intravenous injection of gadopentetate dimeglumine (Magnevist; Schering, Berlin, Germany) with a dosage of 0.1 mmol/kg body weight (TR/TE, 4.8/2.2 ms; FOV, 240 × 240 mm; matrix, 256 × 192 mm; thickness/gap, 5.0/0 mm). Axial and coronal contrast enhanced T1 weighted spin echo sequence with or without fat suppression were also scanned after the intravenous injection of gadolinium contrast medium.

All MRI images from the medical records were blindly and independently verified by two radiologists with over 10 years’ experience on the interpretation of head and neck MRI images. They had to reach an agreement after consultation if their interpretations of the image were inconsistent. The DWI and DCE-MRI data were post-processed using the FuncTool software (GE Healthcare, Chicago, IL, USA). Two radiologists localized the representative regions of interest (ROIs) without bleeding, cystic necrosis, and vessels to measure the ADC value in triplicate and take an average value. The ADC ratio was calculated by dividing the ADC value of the tumor to the ADC value of normal tissue. The tumor thickness was defined as the distance from the tumor surface to the deepest point of invasion. The reference line was drawn along the tumor’s lateral mucosal margin where the tumor appeared largest. The tumor thickness was measured by drawing a perpendicular line from the reference line to the deepest point of tumor extension.

### 2.4. Primary Outcomes and Follow-up

Primary outcomes of interest included the neck recurrence (local recurrence, cervical lymph node metastasis) and the distant metastasis after surgery. Outcomes of all participants were obtained by telephone interviews and data from medical records. The patients were followed-up from the index date until death or the end of the study on 30 April 2021. The disease-free survival (DFS) time was calculated from the index date to the date of recurrence, death, or the end of the study. The overall survival (OS) time was calculated from the index date to the date of death or the end of the study.

### 2.5. Candidate Predictors

The following data were extracted for each patient: age, gender, smoking status, alcohol drinking behavior, number of positive lymph nodes, tumor thickness, ADC value of tumor, ADC value of normal tissue, preoperative pain, preoperative anemia, postoperative anemia, midline crossing, time intensity curve (TIC) shape, preoperative T status and histologic grade. The histologic grades were according to the WHO calcification: I: well-differentiated tumor; II: moderately differentiated tumor; III: poorly differentiated tumor. Surgically excised cervical lymph nodes were carefully examined by the head and neck pathologists for the lymph node-positive (yes/no) and lymph node ratio (LNR), which is defined as the ratio of the number of positive nodes to the total number of dissected nodes. Severity of anemia was classified as mild, moderate, or severe based on the hemoglobin concentration (Hb) in the blood. Surgical treatment consisted of supra-omohyoid, functional or radical. The postoperative adjuvant treatments of radiotherapy, chemotherapy, and concurrent chemo/radiotherapy were also evaluated as yes or no, respectively.

### 2.6. Statistical Analysis

Data were expressed as mean ± SD for normally distributed variables, median (IQR) for non-normally distributed variables and number (percentage) for categorical variables. The normality of the data distribution was examined by using the Kolmogorov-Smirnov tests. Baseline characteristics were compared between patients with and without recurrence by using independent *t*-tests/Mann-Whitney U-Test and the Chi-squared test to detect any differences in the continuous and categorical variables, respectively. Cox proportional hazards model were built to examine the hazard ratio (HR) and 95% confidence intervals (CIs) in different characteristics comparisons. The assumption of proportional hazards was checked. A univariable Cox proportional hazards model was performed first to show the crude effect of each candidate predictors on recurrence. No patients had missing information on any predictors. To develop a multivariable model predicting the recurrence, only predictors that were significant in univariable analyses were included in the model. As a result of the high correlation of the candidate predictors, the set of predictors was further reduced prior to the multivariable analysis. Furthermore, we analyzed time-dependent receiver operator characteristic (ROC) curves of predictors in the multivariable Cox proportional hazards model and calculated the integrated area under the ROC curve (AUC) over time with 95% CI. We also estimated the sensitivity, specificity, maximum value of the Youden index and the optimal cut-off value using ROC curve analysis. DeLong tests were applied to test the difference between the AUCs of different models. The performance of prediction model was evaluated by examining both discrimination and calibration. The area under the ROC curve (AUC) analysis was performed to evaluate discrimination of the model. We also assessed internal validity with a bootstrapping resampling to reduce the over-fitting bias and obtain a realistic estimate of the performance of prediction models. Loess-based calibration plot and the Hosmer-Lemeshow test were used to assess the goodness-of-fit of the model. The Kaplan-Meier method was used to estimate the probability of DFS and OS. The differences between groups were tested with a log-rank test. A nomogram was formulated based on the results of multivariable Cox proportional hazards model by using the package of regression modeling strategies (RMS) in R studio. The concordance index (c-index) was calculated to determine the nomogram’s predictive accuracy for DFS [17]. The nomogram was internally validated using 1000 bootstrap resamples to estimate an unbiased measure of the ability of our predictive model. The calibration curve of nomogram was used to assess the consistency between the predicted DFS and the observed DFS. A two-sided *p*-value of <0.05 was regarded as statistically significant. Data management and statistical analyses were conducted by using SAS version 9.4 software (SAS Institute, Inc., Cary, CA, USA) (proc logistic) and R version 4.1.0 software (rms package, glmnet package, party package, RandomForest package, gbm package, and XGBoost package).

## 3. Results

### 3.1. Baseline Characteristics between the Patients with and without Recurrence

**Figure 2:**
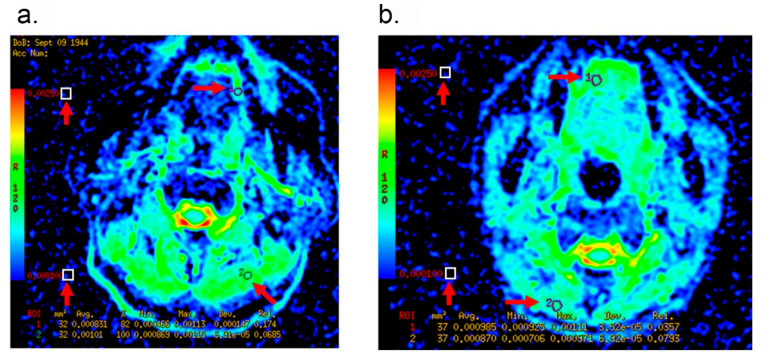
Representative MRI images of patients with cT1-2 OTSCC. Arrows indicate the region of interest (ROI) of the tumor and the non-tumor tissues for ADC measurements. (**a**): A 70-year-old male patient with Recurrence. The ADC value of tumor was measured as 0.831 × 10^−3^ mm^2^/s, and the ADC ratio was 0.822. (**b**): A 41-year-old female patient without Recurrence. The ADC value of tumor was measured as 0.985 × 10^−3^ mm^2^/s, and the ADC ratio was 1.132.

A total of 188 patients met inclusion criteria. Cancer recurrence was seen in 83 patients (44.2%). Of them, neck recurrence, distant metastasis, and neck recurrence plus distant metastasis occurred in 63, 6, and 14 cases, respectively. The mean of the follow-up was 60 months while the median of the follow-up was 69 months (range 2–143 months). As shown in Table 1, the percentage of patients with positive lymph nodes was much higher in patients with recurrence than those without recurrence (59% vs. 34%). Patients with recurrence displayed 200% higher LNR, 60% thicker tumor thickness and 100% more proportion of midline crossing than the cases without recurrence. The ADC values were measured at the regions of interest of the tumor and non-tumor tissues of the MRI images, and the ADC ratio was calculated by dividing the ADC value of the tumor to the ADC value of the adjacent non-tumor tissue as shown in Figure 2. We found that the ADC value of tumor and the ADC ratio in the patients without recurrence were 8% and 17% higher, respectively, compared to the patients with recurrence (Table 1). There was no significant difference of the preoperative T status, the histologic grade, the type of surgery, the postoperative adjuvant treatment and other baseline characteristics between patients with recurrence and those without recurrence.

### 3.2. LNR, Tumor Thickness, ADC Value of Tumor and ADC Ratio Are Candidate Predictors for Prognostic Modeling

To identify the candidate predictors for prognostic modeling, we developed an univariable Cox proportional hazards model (Table 2). We found that the risk factors of lymph node-positive, LNR, tumor thickness, ADC value of tumor, ADC value of normal tissue, and ADC ratio were significantly associated with recurrence. We next introduced the predictors that were significant in univariable analyses into the multivariable Cox proportional hazards model. As a result of the high correlation (Spearman’s correlation coefficient (ρ) = 0.943) between the risk factor of positive lymph nodes and the LNR, we removed the lymph node-positive factor from the model prior to the multivariable model analysis. Since there was a high correlation between the ADC values of normal tissue and the ADC value of tumor or ADC ratio, we also excluded the ADC value of normal tissue from the model. As a result, we selected the LNR, the tumor thickness, the ADC value of tumor, and the ADC ratio as candidate predictor variables for modeling.

### 3.3. Prognostic Model Predicts OTSCC with High Sensitivity and Specificity

**Figure 3:**
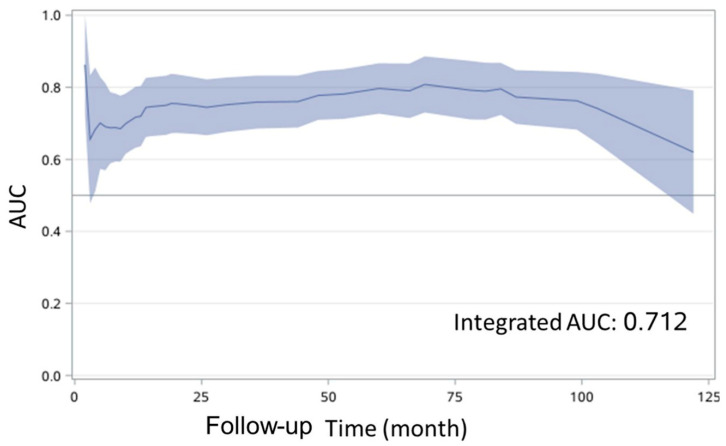
Time-dependent receiver operating characteristic (ROC) curve for disease free survival. AUC, area under the curve.

**Figure 4:**
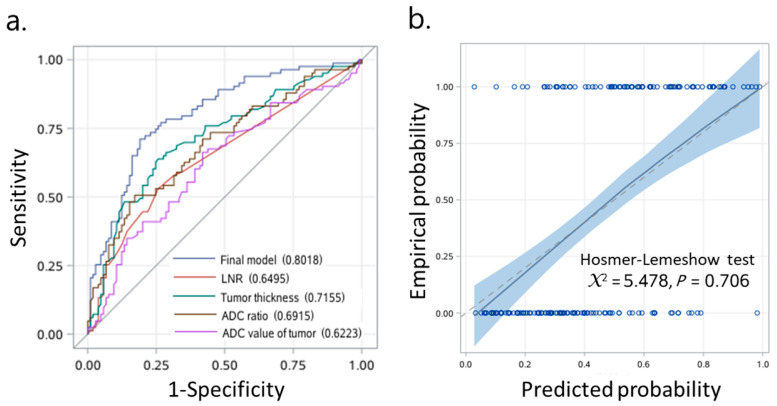
Prognostic model predicts OTSCC outcomes with high sensitivity and specificity. (**a**) Receiver operating characteristic (ROC) curve analysis. (**b**) Calibration plot for disease-free survival. AUC, area under the curve; CI, confidence interval.

To generate a prediction model, we first compared the prognostic performances of three models: a full model with four candidate predictor variables of LNR, tumor thickness, ADC value of tumor and ADC ratio; an ADC model with three candidate predictor variables of LNR, tumor thickness and ADC value of tumor; and an ADC ratio model with three candidate predictor variables of LNR, tumor thickness and ADC ratio. We observed that the full model and the ADC ratio model showed the higher integrated AUC of 0.713 and 0.712, respectively, than that of the ADC model (integrated AUC, 0.663). Next, we included LNR (adjusted HR, 5.57; 95% CI, 0.72–42.79; *p* = 0.099), tumor thickness (adjusted HR, 1.07; 95% CI, 1.03–1.11; *p* < 0.001) and ADC ratio (adjusted HR, 0.09; 95% CI, 0.03–0.26; *p* < 0.001) (Table 2) in the final model. We found that the final model had acceptable discrimination over time, with highest AUC of 0.86 (Figure 3). The AUCs at 1, 3, 5, and 7 years of the follow-ups were 0.72 (95% CI, 0.63–0.80), 0.76 (95% CI, 0.69–0.83), 0.80 (95% CI, 0.73–0.87), and 0.80 (95% CI, 0.72–0.87), respectively. To determine a proper cut-off point, we applied the final model with an individual predictor to generate ROC curves of 4 individual predictors based on logistic regression (Figure 4a). Our analyses from the DeLong test revealed that the AUC of the final model with AUC 0.80 (95% CI, 0.74–0.87) was significantly greater than the AUCs of the other four models. The final model appeared highly sensitive (71.1%) and specific (81.0%). In internal validation, the bootstrap-corrected AUC was 0.79. Examinations of the calibration plot and Hosmer-Lemeshow test revealed that the final model was acceptable, well-calibrated, and feasible (Figure 4b). More importantly, we found that the ADC ratio with AUC 0.69 (95% CI, 0.61–0.77) was superior to the ADC value of the tumor (AUC, 0.62; 95% CI, 0.54–0.70) in the prognostic performance. The optimal cut-off value of LNR, tumor thickness and ADC ratio were 0.04, 12.8 mm and 0.92, respectively.

### 3.4. ADC Ratio-Based Prognostic Model Reliably Predicts OS and DFS of Patients with OTSCC

**Figure 5:**
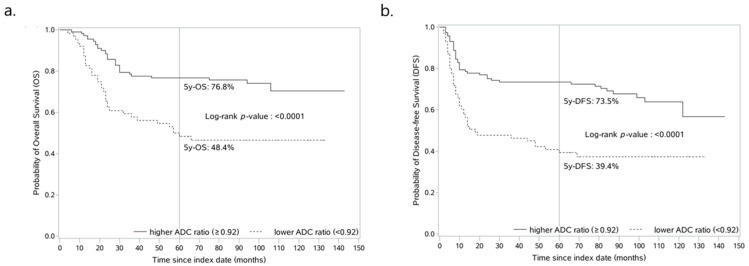
ADC ratio-based prognostic model reliably predicts OS and DFS of patients with OTSCC. Kaplan-Meier curves for estimating the probability of (**a**) overall survival and (**b**) disease-free survival in patients with higher ADC ratio (≥0.92) vs. lower ADC ratio (<0.92). ADC, apparent diffusion coefficient.

The optimal cut-off value of ADC ratio (0.92) produced significant discrimination for better and worse prognosis group in terms of OS (log-rank test, *p* < 0.001, Figure 5a) and DFS (log-rank test, *p* < 0.001, Figure 5b). In patients classified as having a higher ADC ratio (≥0.92, *n* = 117, 62%) and a lower ADC ratio (<0.92, *n* = 71, 38%), the OS rates were 77.7% and 57.8% at 3 years, 76.8% and 48.4% at 5 years, and 70.4% and 46.6% at 10 years of follow-up, respectively (Figure 5a). In addition, the 3-, 5- and 10-year DFS for patients with a higher ADC ratio and a lower ADC ratio were 73.5% and 46.5%, 73.5% and 39.4%, and 63.9% and 37.4%, respectively (Figure 5b).

### 3.5. Nomogram Predicts DFS Reliably and Predictive Accuracy Was Internally Validated

**Figure 6:**
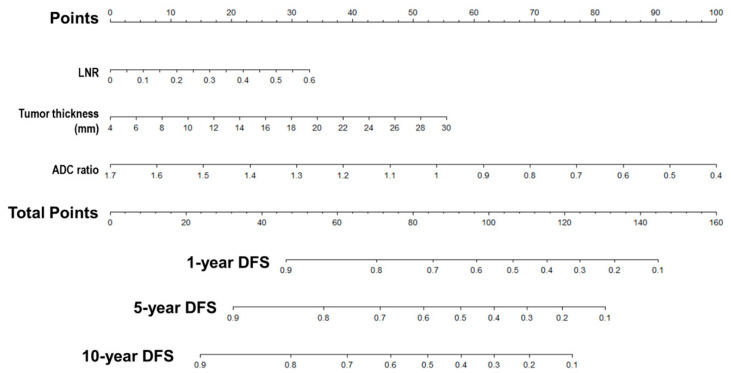
Nomogram predicts DFS reliably and predictive accuracy is internally validated. A nomogram for Disease-Free Survival Prediction. LNR, lymph node ratio; ADC, apparent diffusion coefficient; DFS, disease-free survival.

**Figure 7:**
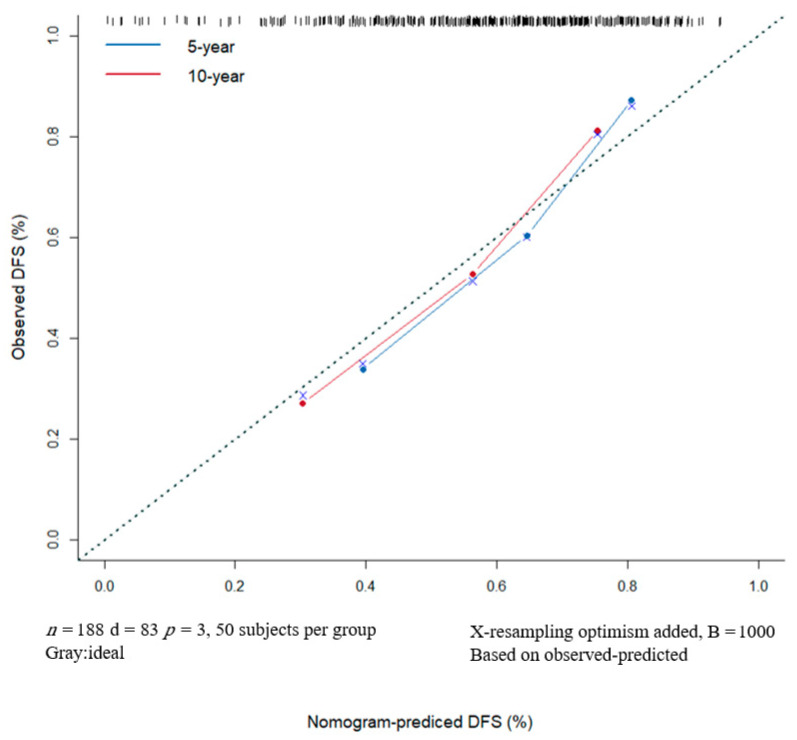
The DFS of 5- and 10-years predicted by the nomogram agrees with the actual observation. Nomogram Model Calibration Curve of 5- and 10-year Disease-free Survival. DFS, disease-free survival.

A nomogram for 1-, 5- and 10-year DFS predictions was developed based on the findings of the multivariable Cox proportional hazards (Figure 6). For the risk factor of ADC ratio, the strongest variable with the greatest effect was assigned 100 points. The rest of the variables (LNR and tumor thickness) were assigned a smaller number of points based on their absolute value of the estimated regression coefficients. Each point of predictor could be obtained by drawing a vertical line from predictor line to the point line. By adding the points from all of the predictors, we had a total of points which corresponded to the predicted probability of 1-, 5- and 10-year DFS. Our study found that the C-index of the nomogram was 0.71 (95% CI, 0.65–0.76). After bootstrapping with 1000 resamples, the corrected C-index was 0.709, indicating a good discriminatory performance of the model. The calibration curve showed that the DFS of 5- and 10-years predicted by the nomogram agreed with the actual observation (Figure 7).

## 4. Discussion

In this study, we developed and internally validated an ADC ratio-based prognostic model for the prediction of OTSCC outcomes. After adjusting lymph node ratio and tumor thickness, we found that the ADC ratio was an independent predictor of cancer recurrence and DFS, and ADC ratio value was superior to the ADC value in the performance of prognostic model. Our study indicates that the prognostic model generated from the ADC ratio value of the tumor is more accurate to predict the OTSCC recurrence and DFS than the model integrated with absolute ADC value, as evidenced in prostate cancer [18,19]. To our knowledge, this study firstly uses the pre-treatment ADC ratios to assess postoperative recurrence and DFS in OTSCC, which provides a useful and efficient clinical predictive tool in practice and helps the clinicians to make and change the therapy for OTSCC patients. Our findings also suggested that in clinical practice, it should increase the postoperative monitoring of patients, and timely provide more accurate and active adjuvant therapy after surgery to improve patients’ DFS and postoperative prognosis when the ADC radios were small.

In agreement with the findings from previous studies on tongue cancer and HNSCC, we found that the pre-treatment ADC value of the tumor was not an important predictor of recurrence [20,21]. However, our findings were not supported by other studies which showed a statistically significant correlation between pre-treatment ADC value of HNSCC and poor prognosis [9,10,11,12,13]. The significant difference of the findings between our study and some of the others could be explained by following reasons. (1) Sample sizes, tumor sites and patient populations were different. We recruited 188 patients with primary cT1-2 of the OTSCC whereas others enrolled less than 100 cases with oropharyngeal squamous cell carcinoma from multiple head and neck sites [22]. (2) The optimal cutoff point of ADC value of tumor for differentiating high- from low-risk HNSCC vary among different studies: 0.79 × 10^−3^ mm^2^/s, 1.21 × 10^−3^ mm^2^/s, and 0.94 × 10^−3^ mm^2^/s [9,23,24]. A systematic review from 12 studies indicated that the prediction of locoregional failure of HNSCC based on the pre-treatment ADC value of tumor were inconsistent, and the sensitivities varied from 50% to 100% because the ADC values could depend on the patient’s characteristics and radiologist’s technical issues [14,25,26,27]. In this study, the optimal cutoff value of ADC ratio was 0.92. The use of the ADC values of normal tissue to normalize the ADC value of tumors has been anticipated to overcome this variation [28]. The risk of postoperative recurrence would be reduced and DFS would be improved when the preoperative ADC ratios were greater than 0.92 in patients with clinically early OTSCC; whereas recurrence risk would be increased when the ADC ratio was less than 0.92, suggesting a necessary of postoperative monitoring of patients and adjuvant therapy after surgery.

Consistent with previous reports that tumor thickness could be used to predict cervical lymph node metastasis in tongue cancer, our study also suggested that tumor thickness was an important predictor of OTSCC recurrence [29,30]. This is not surprising because the deep invading tumors are closer to deep lymphatics and blood vessels, and thus spread to regional lymph nodes [31]. We found the optimal cut-off values for tumor thickness were greater than 12.8 mm, which was close to the best cutoff values observed in another study [20]. High LNR were also reported to be associated with poor survival and high disease recurrence rates in tongue cancer [32,33,34]. It was found superior to traditional TNM lymph node staging in term of the prognostic prediction because it included the information on the extent of cancer spread to the neck (number of positive lymph nodes) and the scope of clearance control (total number of lymph nodes removed during surgery) [35]. In our study, we also showed a significant prognostic impact of LNR in patients with OTSCC, with a similar cut-off value to others [36,37].

Nomograms have become widely used in the prognostic determination of several cancers [38,39,40]. They could help clinicians identify those patients that benefit from extensive surgery and adjuvant treatments as well as more appropriate follow-up care and monitoring. Currently, some nomograms have been reported to predict the recurrence of tongue cancer with a C-index range from 0.58, indicating a fair performance, to 0.72, indicating a good model [33,41]. However, no studies have been performed based on ADC ratio as a risk factor. In this study, we established and internally validated a nomogram that included ADC ratio, tumor thickness and LNR to improve prognostic prediction in OTSCC patients. Our generated nomogram showed satisfactory and reliable discriminative performance with a high value of C-index, AUC, and good calibration. The main advantage of our nomogram was the ability to estimate individualized 5-year and 10-year DFS based on three predictors.

There exist several limitations in this study. First, our prognostic models were not validated by an external data set although the bootstrap-adjusted C-index for internal validation of our nomogram suggested a sufficient level of accuracy that was comparable with that of other published predictive nomograms for tongue cancer [41,42]. We plan to validate our prognostic model with a large size of test samples in future studies. Second, this retrospective cohort included only patients who underwent surgical resection for OTSCC. Patients with unresectable tumors or those who refused surgery were excluded. A prospective study is needed to validate the models. Third, our prognostic models could be improved by integrating more biochemical and/or genomic biomarkers in the model with larger size of patient samples in future. It will be more interesting to evaluate the study method with other pathologies of the same district. While the depth of invasion is a well-recognized predictive factor for OSCC, this histological parameter unfortunately was not analyzed in this study. We will include the depth of tumor invasion in our future study. Forth, the final staging after the surgery and the tumor size on surgery were not included or analyzed as part of multivariate analysis per lack of digital documentation. Finally, our center began to include the HPV infection inspection according to the 8th version of AJCC TNM classification (2017) in 2018, this retrospective study only included the oral tongue squamous cell carcinoma patients without the information on HPV infection from 2009 to 2016.

## 5. Conclusions

In conclusion, a nomogram generated based on ADC ratios was able to predict 1-, 5- and 10-year recurrence risk of OTSCC and help to develop therapeutic strategies for OTSCC treatment. The individual prognostic model built from the ADC ratio value performed better and distinguished the patients with recurrence from those without recurrence more accurately than the one constructed from the conventional ADC value of tumor.

## Figures and Tables

**Table 1:** Baseline and Treatment Characteristics of Patients (Without recurrence vs. with recurrence).

Characteristic	Without Recurrence (*n* = 105)	With Recurrence (*n* = 83)	*p*-Value
Age, years Male sex	51.01 ± 12.11 63 (60.00%)	54.05 ± 13.21 56 (67.47%)	0.102 0.291
Smoking	31 (29.52%)	34 (40.96%)	0.101
Alcohol drinking	23 (21.90%)	24 (28.92%)	0.270
Lymph node-positive	36 (34.29%)	49 (59.04%)	**0.001**
Lymph node ratio (LNR)	0 (0.04)	0.04 (0.12)	**<0.001**
Tumor thickness, mm	10.40 (4.60)	14.30 (7.60)	**<0.001**
ADC value of tumor, ×10^−3^ mm^2^/s	0.98 ± 0.21	0.90 ± 0.23	**0.011**
ADC values of normal tissue, ×10^−3^ mm^2^/s	0.96 (0.41)	1.01 (0.40)	0.107
ADC ratio	1.03 ± 0.21	0.88 ± 0.23	**<0.001**
Preoperative pain	80 (76.19%)	70 (84.34%)	0.167
Preoperative anemia			0.193
No	100 (95.24%)	74 (89.16%)	
Mild	5 (4.76%)	8 (9.64%)	
Moderate	0	1 (1.20%)	
Postoperative anemia			0.198
No	66 (62.86%)	43 (51.81%)	
Mild	38 (36.19%)	37 (44.58%)	
Moderate	1 (0.95%)	3 (3.61%)	
Midline crossing	13 (12.38%)	20 (24.10%)	**0.036**
Time intensity curve (TIC) shape			0.414
Type I	15 (14.29%)	14 (16.87%)	
Type II	85 (80.95%)	68 (81.93%)	
Type III	5 (4.76%)	1 (1.20%)	
Preoperative T status			0.313
cT1	39 (37.14%)	25 (30.12%)	
cT2	66 (62.86%)	58 (69.88%)	
Histologic grade			0.182
I	14 (13.33%)	5 (6.02%)	
I-II	57 (54.29%)	38 (45.78%)	
II	29 (27.62%)	33 (39.76%)	
II-III	4 (3.81%)	6 (7.23%)	
III	1 (0.95%)	1 (1.20%)	
Treatment characteristics			
Type of surgery			0.053
Supra-omohyoid	69 (65.71%)	40 (48.19%)	
Functional	30 (28.57%)	35 (42.17%)	
Radical	6 (5.71%)	8 (9.64%)	
Postoperative adjuvant treatment			0.350
No Radiotherapy (RT)	37 (35.24%) 44 (41.90%)	20 (24.10%) 42 (50.60%)	
Chemotherapy Chemotherapy plus RT	4 (3.81%) 20 (19.05%)	2 (2.41%) 19 (22.89%)	

Data are presented as mean ± SD, median (IQR) or *n* (%). Significant values are showing in bold. Abbreviations: ADC, apparent diffusion coefficient.

**Table 2:** Crude and Adjusted Hazard Ratios for Recurrence in Different Clinical Characteristics Comparisons.

**Variables**	**Crude HR** **(95% CI)**	***p*-Value**	**Adjusted HR ^a^** **(95% CI)**	***p*-Value**
Age, years	1.01 (0.99–1.03)	0.173		
Male sex	1.18 (0.75-1.88)	0.463		
Smoking	1.34 (0.86–2.07)	0.195		
Alcohol drinking	1.22 (0.76–1.97)	0.412		
Lymph node-positive	2.03 (1.31–3.15)	**0.001**		
Lymph node ratio (LNR)	20.44 (3.31–126.43)	**0.001**	5.57 (0.72–42.79)	0.099
Tumor thickness, mm	1.09 (1.05–1.13)	**<0.001**	1.07 (1.03–1.11)	**<0.001**
ADC value of tumor, ×10^−3^ mm^2^/s	0.30 (0.11–0.79)	**0.015**		
ADC values of normal tissue, ×10^−3^ mm^2^/s	2.45 (1.08–5.53)	**0.032**		
ADC ratio	0.07 (0.02–0.21)	**<0.001**	0.09 (0.03–0.26)	**<0.001**
Preoperative pain	1.49 (0.83–2.71)	0.182		
Preoperative anemia				
Mild (vs. No)	1.64 (0.78–3.39)	0.186		
Moderate (vs. No)	3.58 (0.49–26.02)	0.206		
Postoperative anemia				
Mild (vs. No)	1.24 (0.79–1.92)	0.343		
Moderate (vs. No)	3.24 (1.00–10.51)	0.050		
Midline crossing	1.64 (0.99–2.71)	0.054		
Time intensity curve (TIC) shape				
Type II (vs. Type I)	0.86 (0.48–1.53)	0.622		
Type III (vs. Type I)	0.27 (0.04–2.09)	0.211		
Preoperative T status				
cT2 (vs. cT1)	1.15 (0.71–1.83)	0.560		
Histologic grade				
I-II (vs. I)	1.53 (0.60–3.89)	0.372		
II (vs. I)	2.30 (0.89–5.90)	0.082		
II-III (vs. I)	2.53 (0.77–8.28)	0.126		
III (vs. I)	2.94 (0.34–25.15)	0.325		
Treatment characteristics				
Type of surgery				
Functional (vs. Supra-omohyoid)	1.60 (1.02–2.52)	**0.042**		
Radical (vs. Supra-omohyoid)	1.72 (0.80–3.67)	0.165		
Postoperative adjuvant treatment Radiotherapy (RT) (vs. No)	1.38 (0.81–2.35)	0.232		
Chemotherapy (vs. No) Chemotherapy plus RT (vs. No)	0.95 (0.22–4.08) 1.46 (0.78–2.74)	0.950 0.235		

Significant values are showing in bold. ^a^ Adjusted for LNR, tumor thickness, ADC values of normal tissue, ADC ratio, postoperative anemia, type of surgery, and comprehensive treatment. Abbreviations: HR, hazard ratio; CI, confidence interval; ADC, apparent diffusion coefficient.

## Data Availability

The datasets used and/or analysed during the current study are available from the corresponding author on reasonable request.
